# *Iridis tectori* Rhizome Alleviates LPS-Triggered Inflammatory Responses Through Inhibiting NF-κB Signaling in Macrophages

**DOI:** 10.3390/biomedicines14061291

**Published:** 2026-06-05

**Authors:** Yi-Lin Guo, Wen-Jing Li, Xin Huang, Yu-Lin Lin, Yu Liu, Min Cai, Qian Chen, Mu-Qing Wang, Cong-Yu Wu, Yuan Gao, Yun Qi

**Affiliations:** State Key Laboratory of Bioactive Substance and Function of Natural Medicines, Institute of Medicinal Plant Development, Chinese Academy of Medical Sciences & Peking Union Medical College, Beijing 100193, China; pumcxyfwt@163.com (Y.-L.G.); cpuwjli@163.com (W.-J.L.); hx021124@163.com (X.H.); yllin@implad.ac.cn (Y.-L.L.); liuyu9985@126.com (Y.L.); caiminsdu@163.com (M.C.); cq18810621675@163.com (Q.C.); 18353798604@163.com (M.-Q.W.); wucongyu2021@163.com (C.-Y.W.)

**Keywords:** *Iris tectorum* Maxim., Chuan She Gan, inflammation, NF-κB, macrophages, endotoxemia, NO production, iNOS expression

## Abstract

**Objectives:** The rhizome of *Iris tectorum* Maxim. (Chuan She Gan), commonly used as a substitute for She Gan in Sichuan and other regions, is traditionally applied for inflammation-related disorders. This study aimed to evaluate the anti-inflammatory activity of Chuan She Gan ethanolic extract (CSG) and elucidate its molecular mechanism. **Methods:** CSG was prepared by 85% ethanol extraction and analyzed by HPLC to identify representative constituents. In LPS-stimulated RAW264.7 macrophages, nitrite accumulation and iNOS activity, cytokine production and inflammatory gene expression were evaluated using Griess assays, ELISA, and *q*RT-PCR, respectively. NF-κB and AP-1/MAPK signaling were determined using luciferase reporter assays and Wwestern blotting. Serum inflammatory cytokine levels of endotoxemic mice were measured by ELISA. **Results:** Four characteristic isoflavones/glycosides in CSG were identified, including tectoridin, iridin, tectorigenin, and irigenin. In LPS-activated RAW264.7 macrophages, CSG not only dose-dependently suppressed supernatant NO by inhibiting iNOS activity and downregulating iNOS expression, but also reduced IL-6, MCP-1, and intracellular pro-IL-1β at the protein and mRNA levels. Mechanistic analyses indicated that CSG attenuated NF-κB activation by reducing IκBα phosphorylation and limiting p65 nuclear accumulation, while AP-1/MAPK signaling remained largely unchanged. In endotoxemic mice, a single oral gavage of CSG (50–200 mg/kg) significantly lowered serum IL-6, MCP-1, and TNF-α levels. **Conclusions:** CSG showed anti-inflammatory activity in LPS-stimulated macrophages and endotoxemic mice. In LPS-stimulated macrophages, CSG suppressed inflammatory mediator production primarily through the inhibition of NF-κB signaling. In endotoxemic mice, CSG reduced the serum levels of pro-inflammatory cytokines. These findings provide pharmacological basis for the traditional use of Chuan She Gan.

## 1. Introduction

Inflammation is an essential component of host defense, orchestrating the clearance of invading pathogens and the initiation of tissue repair. Macrophages are widely distributed sentinel cells that play a vital protective role in the early stage of inflammation. They can recognize danger signals through pattern-recognition receptors (PRR) and rapidly produce inflammatory mediators, thereby shaping immune responses [1]. Among diverse stimuli, lipopolysaccharide (LPS), a major pathogen-associated molecular pattern (PAMP) from Gram-negative bacteria, is sensed by its PRR toll-like receptor 4 (TLR4) on macrophages to initiate type 1 inflammatory responses [2]. Engagement of the LPS (PAMP)-TLR4 (PRR) axis triggers recruitment of adaptor proteins such as MyD88 and subsequently activates multiple downstream signaling cascades, thereby activating nuclear factor-κB (NF-κB) and activator protein-1 (AP-1), two major transcriptional regulators of inflammatory responses [3].

Under basal conditions, NF-κB (p50/p65) dimers are retained in the cytoplasm through association with an inhibitor of κB (IκB) proteins. After inflammatory stimulation, activated IκB kinase (IKK) phosphorylates IκBα promotes its ubiquitination and degradation, thereby releasing p50/p65, allowing its nuclear translocation and transcriptional regulation of genes such as iNOS, IL-6, MCP-1, and IL-1β [4,5]. Another transcriptional route involves MAPK-dependent regulation of AP-1, with JNK, ERK1/2, and p38 serving as major upstream kinases [6]. However, when macrophages are persistently activated, the excessive release of these pro-inflammatory mediators can amplify inflammatory cascades, leading to tissue injury, organ dysfunction, and even driving the onset and progression of various acute and chronic inflammation-related diseases [7]. Accordingly, targeting macrophage hyperactivation and excessive mediator release is a rational strategy for controlling inflammation-related pathology.

*Iris tectorum* Maxim. (Iridaceae) (ITM) is a perennial ornamental species indigenous to China. It was recorded in the first Chinese monograph on herbal medicines “*Shennong Bencao Jing*” (A.D. 69–100). The rhizome of *Iris tectorum*, known as “Yuan Gen” in Chinese, is the main medicinal part and was historically used for removing “zhengjia jieju”, expelling parasites, as well as detoxifying the body. *Shennong Bencao Jing* also records another herb from the same family (Iridaceae)—“She Gan”, the rhizome of *Belamcanda chinensis* (L.) DC. (BC). It was mainly used for the treatment of upper respiratory tract infection-related disorders, such as sore throat, productive cough, shortness of breath and pyrexia, which are markedly different from the traditional uses of “Yuan Gen”. Maybe due to their high similarity during the non-flowering period, the rhizome of ITM was often mistakenly used as BCs. By the Ming Dynasty, Li Shizhen included this practice in his “*Compendium of Materia Medica*” (A.D. 1578), turning a previous misconception into an accepted norm [8]. Modern studies also indicate that both BC and ITM share similar composition, including isoflavones, flavonoids, volatile oils, and benzoquinones, etc. [9,10,11]. In Sichuan province, a major producing area of ITM, “Yuan Gen” (referred to as “Chuan She Gan”) has been mainly used for treating respiratory tract inflammatory diseases, similar to “She Gan” [12].

In fact, “She Gan” was incorporated into the Chinese Pharmacopoeia as early as 1963 and has more recently been included in the European Pharmacopoeia [13]. By contrast, “Chuan She Gan” was not included in the Chinese Pharmacopoeia until 2005 [14]. According to the latest edition [15], it is used for relieving sore throat, dispersing phlegm, as well as heat-clearing and detoxifying, which is identical to those of “She Gan” [16]. In contrast to the well-studied “She Gan” [9,17,18], this study focused on the understudied “Yuan Gen” and investigated its effects and molecular mechanism(s) using the lipopolysaccharide (LPS)-induced M1 macrophages and endotoxemia mice.

## 2. Material and Methods

### 2.1. Reagents

Fetal bovine serum (Cat No. 11011-8611) was purchased from Zhejiang tianhang Biotechnology Co. (Huzhou, China). DMEM (Cat No. 12100-046) was obtained from Gibco BRL (Grand Island, NY, USA). Dexamethasone (Cat No. S17003) and albumin (bovine serum; Cat No. S12012) were obtained from Yuanye Biotechnology Co. (Shanghai, China). L-NAME (Cat No. GA11233) was from Glpbio Technology Co. (Montclair, CA, USA). MTS (Cat No. G111A) was from Promega Co. (Madison, WI, USA). LPS (Cat No. L4130) was from Sigma-Aldrich (St. Louis, MO, USA). Mouse IL-6 (Cat No. 431301), MCP-1 (Cat No. 432701) and IL-1β (Cat No. 432601) ELISA kits were from BioLegend Co. (San Diego, CA, USA). TRIzol reagent was from Invitrogen Co. (Carlsbad, CA, USA). EasyScript All-in-One First-Strand cDNA Synthesis SuperMix for *q*PCR (One-Step gDNA Removal) (Cat No. AE341-02) and mammalian total protein extraction kit (Cat No. DE101) were acquired from TransGen Biotech Co. (Beijing, China). 2× *q*PCR SYBR Green Master Mix (No Rox) (Cat No. 11201ES08) was from Yeasen Biotechnology Co. (Shanghai, China). Griess reagent nitric oxide assay kit (Cat No. S0021), protease and phosphatase inhibitor cocktail (Cat No. P1050), nuclear and cytoplasmic protein extraction kit (Cat No. P0028), NFκB-TA-luc (Cat No. D2207), AP1-TA-luc (Cat No. D2108) reporter plasmids, One-Lumi™ II Firefly Luciferase Reporter Gene Assay Kit (Cat No. RG056), DEPC water (Cat No. R0021) and prestained color protein marker (Cat No. P0068) were from Beyotime Institute of Biotechnology (Haimen, China). Antibodies against iNOS (Cat No. 13120S), NF-κB p65 (Cat No. 4764), IκBα (Cat No. 4814), p-IκBα (Cat No. 2859), p-JNK (Cat No. 9251), p-ERK (Cat No. 4370), p-p38 (Cat No. 9215) were obtained from Cell Signaling Technology (Danvers, MA, USA). Mouse TNF-α high sensitivity ELISA kit (Cat No. RK04875), antibodies for GAPDH (Cat No. AC033) and vinculin (Cat No. A2752), horseradish peroxidase (HRP)-conjugated anti-mouse (Cat No. AS003), and anti-rabbit (Cat No. AS014) IgG were from ABclonal Biotech Co. (Wuhan, China). BCA Protein Assay Kit (Cat No. CW0014S) and SDS-PAGE Loading Buffer (Cat No. CW0027S) were obtained from Cwbio Biotechnology Co. (Taizhou, China). Ultra-sensitive ECL luminescence substrate (Cat No. BL520A) was from Labgic Technology Co. (Beijing, China). Tectoridin (Cat No. BP1368, purity ≥ 98%) was purchased from Chengdu Biopurify Phytochemicals Co. (Chengdu, China). Irigenin (Cat No. PS2206-0020, purity ≥ 98%) was from Chengdu Push Bio-technology Co. (Chengdu, China). Iridin (Cat No. B21480, purity ≥ 98%) was purchased from Shanghai Yuanye Bio-Technology Co. (Shanghai, China), and tectorigenin (Cat No. A0201, purity ≥ 98%) was purchased from Chengdu Must Bio-Technology Co. (Chengdu, China).

### 2.2. Cells Culture and Treatment

RAW264.7 cells were obtained from American Type Culture Collection (No. TIB-71; ATCC, Manassas, VA, USA). They were maintained in a humidified incubator at 37 °C with 5% CO_2_ and cultured in DMEM complete medium supplemented with 10% fetal bovine serum (FBS), 100 U/mL penicillin and 100 μg/mL streptomycin.

### 2.3. Animals

The male ICR mice (SPF grade, 20 ± 2 g) were obtained from Vital River Experimental Animal Services (Beijing, China; license number: SYXK (Beijing) 2023-0008). Animals were housed in a specific pathogen-free facility under a 12 h light/12 h dark cycle with controlled temperature and humidity.

All experimental procedures were approved and supervised by the Institutional Animal Care and Use Committee of the Institute of Medicinal Plant Development (IMPLAD), Chinese Academy of Medical Sciences & Peking Union Medical College. All animal experiments in this study were conducted in strict accordance with the *Guide for the Care and Use of Laboratory Animals (ethical approval number SLXD-20251215011)*. In addition, anesthesia with isoflurane and euthanasia was performed to minimize animal suffering during the procedures.

### 2.4. Preparation of CSG

The *Iris tectorum* Maxim. was collected in the Medicinal Plant Garden of the Institute of Medicinal Plant Development (IMPLAD), Chinese Academy of Medical Sciences and identified by Yu-Lin Lin. The authenticated specimen was archived in IMPLAD with the voucher code 2024-CSG-369. The rhizomes were washed, sliced, and oven-dried. Subsequently, dried samples were soaked overnight in 85% ethanol (1:20, w/v), followed by sufficiently refluxing twice, 2 h each time. After filtration, the combined ethanolic extract was evaporated under reduced pressure using an N-1100 rotary evaporator (EYELA, Tokyo, Japan), allowing ethanol recovery. The concentrate was further evaporated to a constant weight. Extraction yield was calculated to be 30.33% (w/w). For cell-based assays, CSG was dissolved in dimethyl sulfoxide (DMSO), with the final DMSO concentration maintained below 0.4% in the culture medium. For gavage, CSG was suspended in 0.5% CMC-Na solution.

### 2.5. HPLC Analysis

Chemical profiling and identification of CSG were performed using high-performance liquid chromatography (HPLC). The analysis was performed using an LC-15C HPLC system equipped with LC solution software version 1.26 SP1 and a UV detector (Shimadzu, Japan). Chromatographic separation was achieved on a reversed-phase Syncronis™ C18 column (4.6 mm × 250 mm, 5 μm; Thermo Fisher Scientific, Waltham, MA, USA). The mobile phase consisted of acetonitrile (A) and 0.2% formic acid (B). The gradient elution program was as follows: 0–10 min 10–30% A; 10–40 min 30–50% A; 40–50 min 50–60% A; 50–70 min 60–10% A. Chromatographic detection was performed at 265 nm with a mobile-phase flow rate of 1.0 mL/min. Components in CSG were identified by comparing their retention time with that of authentic reference standards, and quantification was performed using the external standard method.

### 2.6. Endotoxemia Mouse Model

ICR mice were randomly divided into five groups, including a control group, a model group, and three CSG treatment groups. After overnight fasting, mice in the CSG treatment groups were, respectively, administered CSG by oral gavage (i.g.) at 50, 100, and 200 mg/kg, while mice in other groups were given the same volume of CMC-Na. After 30 min, endotoxemia was induced by tail-vein administration of LPS at 1 mg/kg, with saline used for the control group. Two hours after injection, blood samples were collected and kept at 4 °C overnight. ELISA kits were used to determine cytokine levels in serum, with quantification performed against assay-specific standard curves.

### 2.7. Cytotoxicity Assay

Cell viability after CSG exposure was determined in RAW264.7 cells using an MTS-based method. RAW264.7 macrophages were seeded into 96-well plates at a density of 8 × 10^3^ cells per well and incubated with CSG at various concentrations (25, 50, 100, 200, and 400 μg/mL) for 22 h. Subsequently, 0.5% MTS solution (20 μL/well; MTS:PMS = 20:1) was added and followed by an additional 2 h incubation. Absorbance was recorded at 492 nm using a Multiskan Ascent 354 microplate reader (Thermo Fisher Scientific Inc., Waltham, MA, USA).

### 2.8. Determination of NO Production

NO production was estimated by measuring nitrite accumulation in the culture supernatant using the Griess reaction [19,20]. RAW264.7 cells were plated in 96-well plates at 4 × 10^5^ cells/well and pretreated with CSG at 50, 100, and 200 μg/mL, or with L-NAME at 80 μM, for 1 h. The cells were then challenged with LPS at 10 ng/mL for 24 h. After stimulation, 100 μL of supernatant from each well was mixed with an equal volume of Griess reagent. Absorbance was recorded at 540 nm using a Multiskan Ascent-354 microplate reader (Thermo Fisher Scientific Inc., Waltham, MA, USA). Nitrite concentrations were calculated from a standard curve.

### 2.9. Assessment of iNOS Activity

Intracellular iNOS activity was evaluated using a nitrite-based method described previously [21]. RAW264.7 cells were first exposed to LPS at 10 ng/mL for 12 h to induce iNOS expression. The LPS-containing medium was then removed and the cells were washed three times with D-Hank’s solution. The preactivated cells were reseeded into 96-well plates at 4 × 10^5^ cells/well and incubated with the indicated concentrations of CSG or L-NAME for another 12 h. Nitrite levels in the culture supernatant were used as the indirect readout of iNOS enzymatic activity.

### 2.10. Measurement of Inflammatory Mediators

For cytokine measurement, RAW264.7 cells were seeded into 96-well plates at 4 × 10^5^ cells/well. Cells were preincubated with CSG or dexamethasone at 20 μM for 1 h, followed by stimulation with LPS at 10 ng/mL for 24 h. IL-6 and MCP-1 were measured in culture supernatants, whereas IL-1β was detected in cell lysates. All mediators were quantified using commercial ELISA kits according to the supplied protocols. Concentrations were calculated from the corresponding standard curves.

### 2.11. Total RNA Extraction and qRT-PCR

RAW264.7 cells were plated in 6-well plates at 1 × 10^7^ cells/well for gene-expression analysis. After 1 h of pretreatment with CSG, cells were stimulated with LPS at 10 ng/mL for 4 h. Total RNA was isolated using Trizol reagent and cDNA was synthesized with TransScript All-in-One First-Strand cDNA Synthesis SuperMix. The *q*RT-PCR was performed on a BIOER Fluorescent Quantitative Detection System using 2× *q*PCR SYBR Green Master Mix. GAPDH was used as the endogenous control. Relative mRNA expression was calculated using the 2^−ΔΔCt^ method. Primer sequences were designed and synthesized by Sangon Biotech Co. (Shanghai, China) and are listed in Table 1.

### 2.12. Plasmids Transfection and Luciferase Reporter Assay

Stable RAW264.7 cells expressing the pNFκB-TA-luc or pAP-1-TA-luc were prepared in clear-bottom white 96-well plates at 4 × 10^5^ cells per well. After 1 h of treatment with CSG at the indicated concentrations, cells were stimulated with LPS at 10 ng/mL for 4 h. Luciferase signals were developed using One-Lumi™ luciferase reporter reagent and recorded with a MicroBeta2 microplate counter (PerkinElmer, Waltham, MA, USA). Reporter activity was calculated relative to the corresponding control group.

### 2.13. Western Blotting

RAW264.7 cells seeded in 6-well plates at 1 × 10^7^ cells/well were pretreated with CSG for 1 h and then stimulated with LPS at 10 ng/mL for the indicated time periods. Total proteins were extracted using a mammalian protein extraction kit supplemented with protease and phosphatase inhibitors. Nuclear and cytoplasmic fractions were prepared with a commercial nuclear and cytoplasmic extraction kit. Protein concentrations were determined by BCA assay, and all samples were adjusted to equal concentrations before electrophoresis. For immunoblotting, 10 μg protein from each sample was separated by 10–12% SDS-PAGE and transferred to PVDF membranes using a semi-dry transfer system. Membranes were blocked with 5% non-fat milk for 2 h at room temperature and then incubated with the indicated primary antibodies overnight at 4 °C. After washing with TBST, membranes were incubated with the corresponding HRP-conjugated secondary antibodies for 1 h at room temperature. Protein bands were detected using ECL substrate and imaged with a Touch Imager system (e-BLOT, Shanghai, China). Densitometric analysis was performed using Image J software version 1.52a (Bethesda, MD, USA), and target protein levels were normalized to the corresponding internal controls.

### 2.14. Statistical Analysis

Data were analyzed using GraphPad Prism 9.0.0 (GraphPad Software, Inc., La Jolla, CA, USA) and are expressed as mean ± SD from at least three independent experiments. An unpaired Student’s *t*-test was applied for two-group comparisons, whereas multiple-group comparisons were analyzed by one-way ANOVA followed by Tukey’s post hoc test. A *p* value below 0.05 was regarded as statistically significant.

## 3. Results

### 3.1. Chemical Characterization of CSG by HPLC

**Figure 1:**
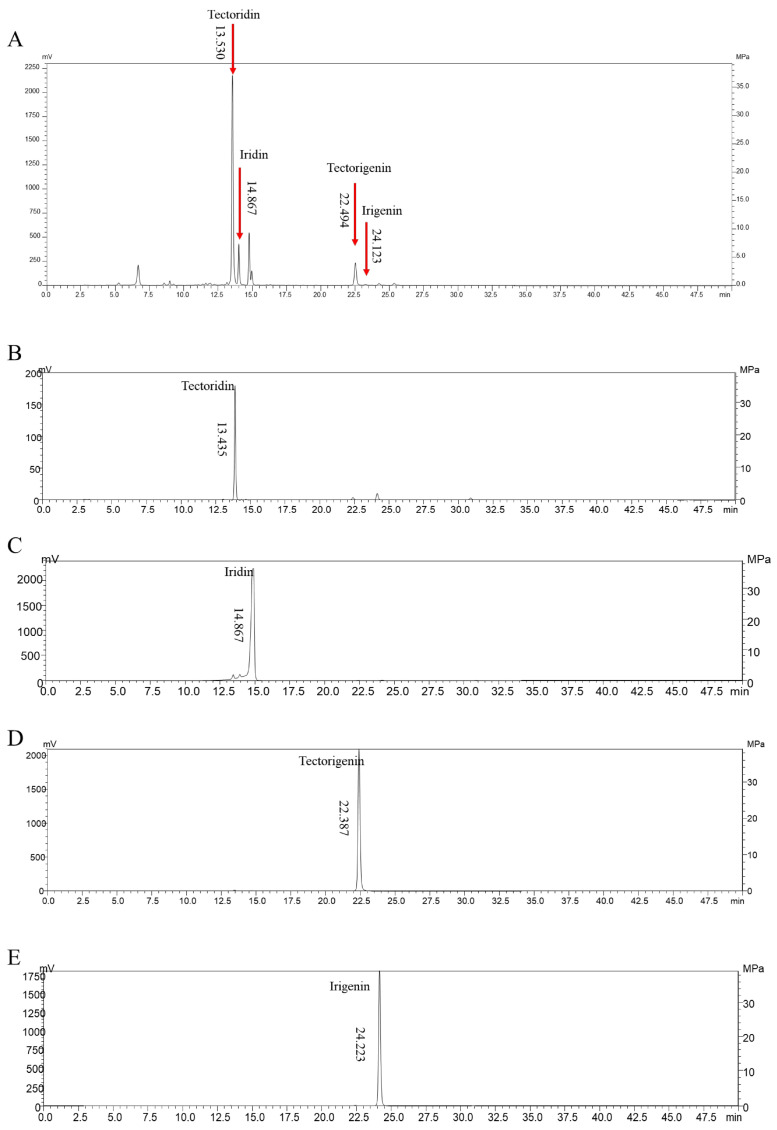
HPLC chromatograms of CSG and standard compounds. (**A**) The fingerprint of CSG. (**B**–**E**) The HPLC chromatograms of tectoridin, iridin, tectorigenin, and irigenin.

Modern phytochemical studies on the rhizomes of *Iris tectorum* [10] and comparative studies involving BC and ITM [9,11] have shown that *Iris tectorum* Maxim. is rich in isoflavonoids, particularly isoflavones and their glycosides. Among them, tectoridin, iridin, and their corresponding aglycones tectorigenin and irigenin have been most extensively reported as characteristic compounds [9,10,11]. The fingerprint of CSG was shown in Figure 1A. Accordingly, we compared the fingerprint of CSG with reference standards of these four compounds. Based on retention time, all four constituents were identified in the fingerprint of CSG (Figure 1B–E). Furthermore, external standard calibration curves were generated for each standard to enable quantitative determination. The results showed that the contents of tectoridin, iridin, tectorigenin, and irigenin in CSG were determined to be 15.38%, 3.75%, 3.70%, and 0.12%, respectively.

### 3.2. CSG Alleviates Pro-Inflammatory Cytokines in Mouse Endotoxemia Model

**Figure 2:**
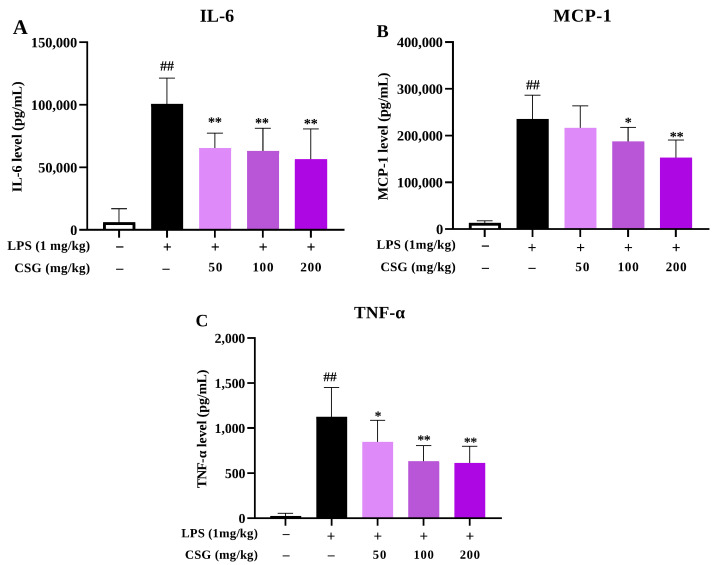
Effect of CSG on serum inflammatory mediators in endotoxemic mice (*n* = 10). CSG was administered by oral gavage 30 min before intravenous LPS challenge. Blood samples were collected 2 h later. Serum IL-6 (**A**), MCP-1 (**B**), and TNF-α (**C**) levels were measured. ^##^ *p* < 0.01 vs. normal control group; * *p* < 0.05 and ** *p* < 0.01 vs. LPS alone group.

An LPS-induced endotoxemia model was established to evaluate the in vivo anti-inflammatory effect of CSG. LPS challenge via the tail vein caused a marked rise in circulating IL-6, MCP-1, and TNF-α within 2 h. When CSG was administered once by oral gavage at 50, 100, and 200 mg/kg 30 min prior to LPS challenge, serum levels of these mediators were significantly reduced in a dose-dependent manner, indicating that CSG also exerts potent anti-inflammatory effects in vivo (Figure 2A–C).

### 3.3. CSG Suppresses LPS-Induced NO Production in Macrophages

**Figure 3:**
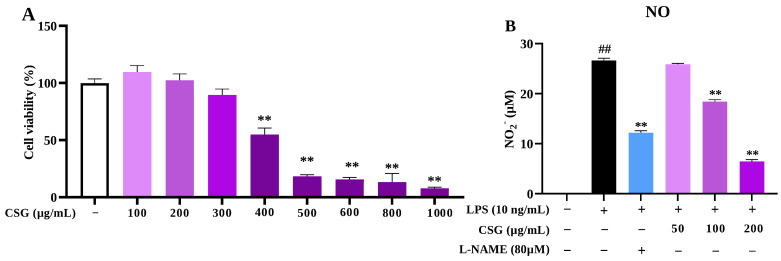
Cytotoxicity evaluation and NO inhibition effects of CSG in RAW264.7 macrophages (*n* = 3). (**A**) Viability of RAW264.7 cells after CSG treatment. (**B**) Supernatant NO levels after LPS stimulation. ^##^ *p* < 0.01 vs. normal control group; ** *p* < 0.01 vs. LPS alone group.

The cytotoxic profile of CSG was initially assessed in RAW264.7 macrophages, where viability remained unchanged at doses not exceeding 200 μg/mL (Figure 3A). Accordingly, three non-cytotoxic doses (50, 100, and 200 μg/mL) were selected for the subsequent in vitro assays. As expected, LPS markedly increased NO release relative to untreated cells, whereas CSG treatment decreased supernatant NO level (Figure 3B).

### 3.4. CSG Inhibits iNOS Expression via Suppressing Its Enzymatic Activity and Transcription

**Figure 4:**
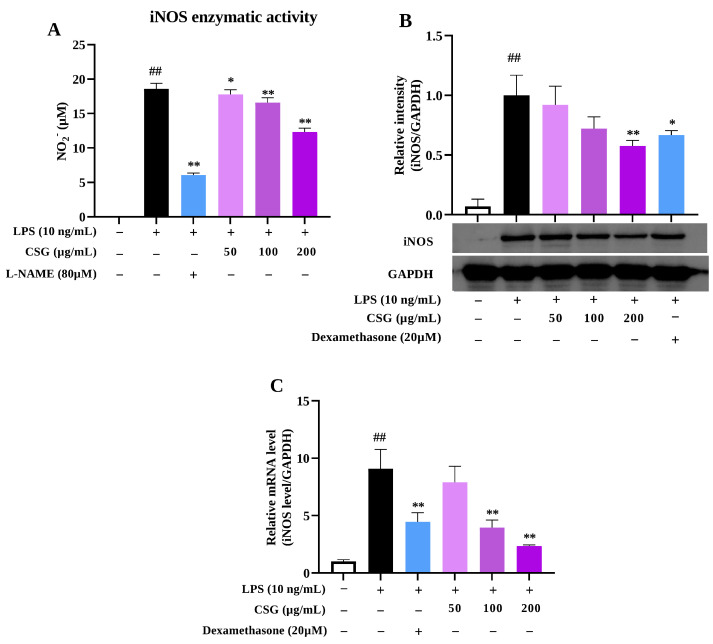
Regulation of iNOS activity and expression by CSG in LPS-stimulated RAW264.7 cells (*n* = 3). (**A**) Effects of CSG on iNOS enzymatic activity. RAW264.7 cells were primed with LPS for 12 h, washed to remove LPS, and then exposed to CSG or L-NAME for another 12 h. Nitrite generation was measured as an indirect readout of iNOS enzymatic activity. (**B**,**C**) Changes in iNOS protein and transcript levels after CSG treatment. ^##^ *p* < 0.01 vs. normal control group; * *p* < 0.05 and ** *p* < 0.01 vs. LPS alone group.

In LPS-stimulated macrophages, iNOS is the major enzymatic source responsible for NO generation. Changes in either the activity or abundance of iNOS can directly influence NO production. In this study, RAW264.7 cells were exposed to LPS for 12 h, after which LPS-free medium was replaced. Since iNOS is an inducible enzyme, no new iNOS is synthesized in the absence of LPS; therefore, the amount of iNOS remains constant, and any alteration in NO level exclusively attributed to the change in enzyme activity [21]. As shown in Figure 4A, CSG lowered NO production in a dose-dependent manner, demonstrating that it can inhibit iNOS activity (Figure 4A). Moreover, Western blotting and *q*RT-PCR analyses showed that CSG also reduced iNOS protein and mRNA levels in LPS-activated macrophages (Figure 4B,C). Together, these results suggest that CSG limits NO production by targeting both iNOS activity and iNOS expression.

### 3.5. CSG Suppresses LPS-Induced Pro-Inflammatory Cytokines

**Figure 5:**
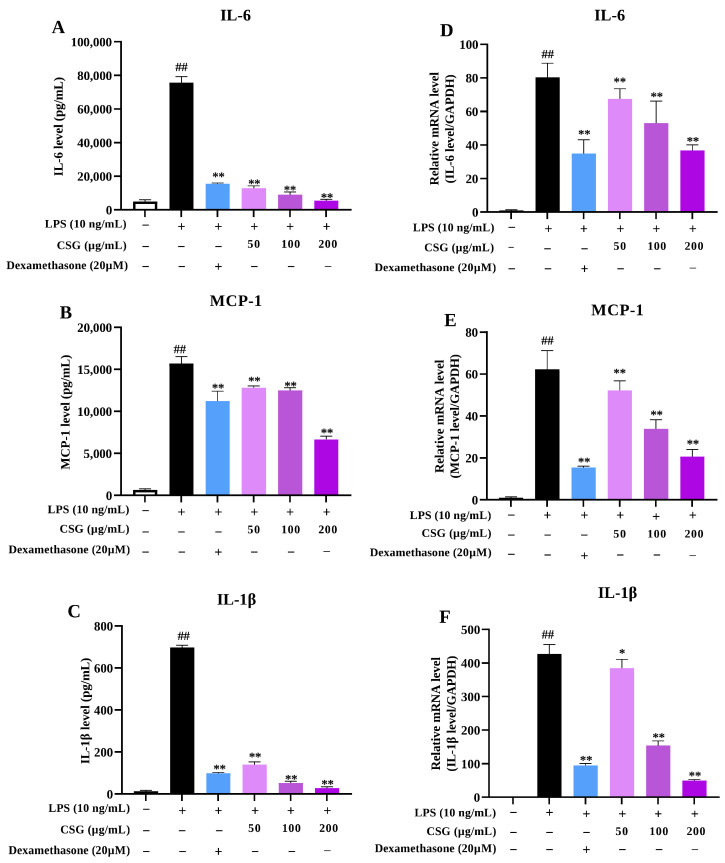
CSG reduced cytokine levels and inflammatory transcripts in LPS-activated RAW264.7 cells. (**A**–**C**) Impact of CSG on supernatant IL-6, supernatant MCP-1, and intracellular pro-IL-1β in cell lysates (*n* = 3). (**D**–**F**) Modulatory effect of CSG on MCP-1, IL-6, and IL-1β mRNA expression (*n* = 4). ^##^ *p* < 0.01 vs. normal control group; * *p* < 0.05 and ** *p* < 0.01 vs. LPS alone group.

IL-6 and MCP-1 were next examined as additional inflammatory mediators released by LPS-stimulated macrophages. CSG markedly lowered IL-6 levels in the supernatant of LPS-treated RAW264.7 cells, with 50 μg/mL producing an inhibition rate of 82.95% (Figure 5A). It also exerted a modest inhibitory effect on MCP-1 (Figure 5B). Given the limited amount of IL-1β released into the supernatant, intracellular pro-IL-1β was used for evaluation [23]. The results demonstrated that CSG also markedly decreased the intracellular pro-IL-1β as well (Figure 5C).

To further determine whether these reductions at the secretory level were accompanied by transcriptional changes, IL-6, MCP-1, and IL-1β mRNA levels were measured by *q*RT-PCR. Consistently, CSG downregulated the mRNA expression levels of these genes in a concentration-dependent manner (Figure 5D–F), indicating that CSG can suppress inflammatory mediator production through transcriptional repression.

### 3.6. CSG Does Not Affect MAPK/AP-1 Signaling

**Figure 6:**
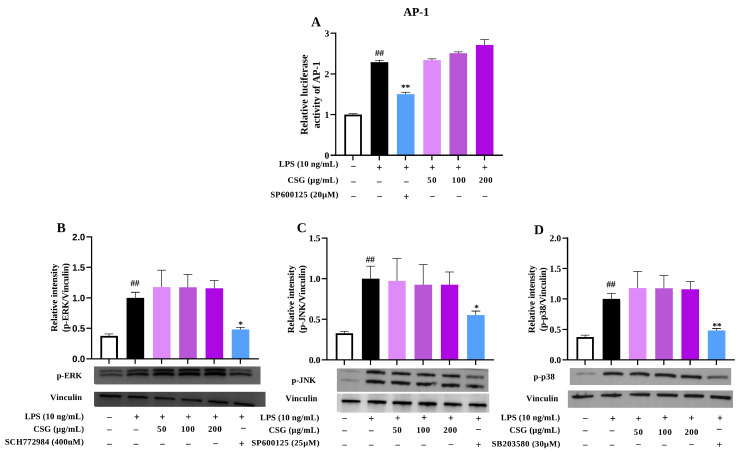
Influence of CSG on AP-1 activation and MAPK phosphorylation in LPS-activated RAW264.7 cells. (**A**) Impact of CSG on AP-1 reporter activity (*n* = 3). (**B**–**D**) Modulatory effect of CSG on LPS-induced phosphorylation of ERK, JNK, and p38 (*n* = 3). ^##^ *p* < 0.01 vs. normal control group; * *p* < 0.05 and ** *p* < 0.01 vs. LPS alone group.

AP-1 participates in the transcriptional regulation of various inflammatory genes, including iNOS, IL-6, MCP-1, and IL-1β [24]. Given that CSG exerted pronounced inhibitory effects on the transcriptional levels of these mediators, we assessed AP-1 transcriptional activity using a luciferase reporter system, which showed that CSG did not significantly alter LPS-induced AP-1 reporter activity across the tested concentrations (Figure 6A).

AP-1 activation is commonly regulated by MAPK signaling, particularly through JNK, ERK1/2, and p38 phosphorylation [24]. To further corroborate our findings, we examined the phosphorylation status of the three major MAPKs that mediate AP-1 activation. Consistently, Western blot analyses demonstrated that LPS stimulation markedly triggered the phosphorylation of JNK, ERK1/2, and p38, while CSG also had no obvious effect on them (Figure 6B–D). These results indicate that MAPK/AP-1 signaling is unlikely to be the pathway affected by CSG in LPS-activated RAW264.7 macrophages.

### 3.7. CSG Significantly Inhibits NF-κB Signaling

**Figure 7:**
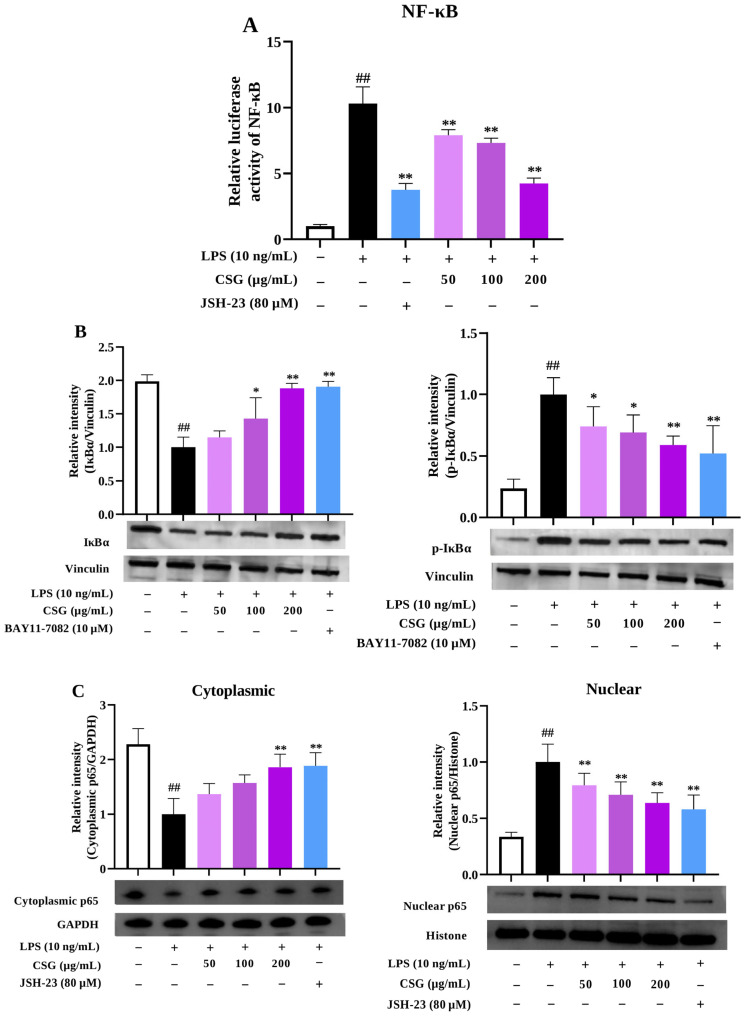
Influence of CSG on NF-κB activation in LPS-activated RAW264.7 cells. (**A**) Impact of CSG on NF-κB reporter activity (*n* = 3). (**B**,**C**) Modulatory effect of CSG on IκBα phosphorylation and p65 nuclear translocation (*n* = 3). ^##^ *p* < 0.01 vs. normal control group; * *p* < 0.05 and ** *p* < 0.01 vs. LPS alone group.

NF-κB is another key transcription factor implicated in LPS-triggered inflammatory responses, whose activation also initiates the transcription of downstream inflammatory mediators mentioned above [4,24]. NF-κB-dependent luciferase activity was therefore measured. As expected, LPS stimulation markedly increased NF-κB reporter activity, whereas CSG dose-dependently suppressed its activation (Figure 7A).

LPS stimulation can lead to the phosphorylation and subsequent degradation of IκBα, thereby facilitating p65 nuclear translocation and the transcription of downstream inflammatory genes [4,25]. Our results showed that IκBα phosphorylation was increased upon LPS stimulation, accompanied by a decrease in p65 in the cytoplasmic and an increase in it in the nuclear. Two positive controls, JSH-23 (the inhibitor for the nuclear translocation of NF-κB p65 [26]) and BAY11-7082 (the inhibitor of IκBα phosphorylation [27]) effectively blocked the corresponding NF-κB activation-related events; and treatment of CSG concentration-dependently reversed these changes (Figure 7B,C).

## 4. Discussion

Modern phytochemical studies on the rhizomes of *Iris tectorum* generally demonstrate that the characteristic constituents of Chuan She Gan are mainly isoflavonoids and their glycosides [10], among which tectoridin, iridin, tectorigenin, and irigenin are the most representative. These constituents had been found to suppress LPS-induced inflammatory activation by decreasing NO release and the expression of pro-inflammatory cytokines [28,29,30,31], suggesting that they may contribute to the main bioactivity-associated constituents of CSG. Therefore, we applied an HPLC method to determine the presence and contents of the four bioactive compounds, among which tectorigenin and irigenin are isoflavonoids, while tectoridin and iridin are the corresponding glycosides (Figure 1). Based on the HPLC quantitative analysis, the contents of the four constituents in CSG were determined to be 15.38%, 3.75%, 3.70%, and 0.12%, respectively. Notably, tectoridin was the most abundant isoflavonoid in CSG, supporting the rationale for its selection as the chemical quality-control marker for Chuan She Gan in the Chinese Pharmacopoeia [15].

Since macrophages are major cellular responders to LPS, we next determined whether CSG could affect pro-inflammatory mediator production in RAW264.7 cells. NO represents a major macrophage-derived pro-inflammatory mediator, whose accumulation after LPS exposure is mainly driven by induction of iNOS. While an appropriate level of NO contributes to host defense, excessive NO production can trigger nitrative stress, promote vasodilation and vascular permeability changes, ultimately lead to tissue injury, thereby contributing to inflammatory pathology in acute and chronic disease contexts [32]. Our data showed that CSG attenuated the LPS-induced NO response without causing detectable cytotoxicity under the tested conditions (Figure 3 and Figure 4B,C). Moreover, CSG also inhibited iNOS enzymatic activity, which may jointly contribute to the decrease in supernatant NO production (Figure 4A).

IL-6, whose elevation is closely associated with inflammatory severity, is a central driver of the acute-phase response and systemic inflammatory amplification [33]. Complementarily, MCP-1 (CCL2) acts mainly as a chemotactic signal that attracts monocytes or macrophages to sites of inflammation, where excessive cell influx can intensify tissue damage [34]. Our results demonstrated that CSG decreased the release of both mediators, especially with an inhibitory rate of up to 82.95% on IL-6 at 50 μg/mL (Figure 5). Since the level of IL-1β in RAW264.7 supernatants was too low to be reliably quantified [23], intracellular pro-IL-1β was assessed instead. CSG markedly decreased its level as well (Figure 5). Consistently, these inhibitory effects were also determined at the transcriptional level, with IL-6, MCP-1, and IL-1β transcripts progressively decreasing as the CSG concentration increased (Figure 5). The parallel decreases in mediator abundance and mRNA expression suggest that CSG interferes with the transcriptional control of macrophage inflammatory activation.

NF-κB and AP-1 are major transcriptional regulators in activated macrophages and control a broad set of inflammatory genes, including iNOS, IL-6, MCP-1, and IL-1β, etc. [24,35]. Accordingly, we further evaluated the effects of CSG on these two signaling. The obtained results revealed that through inhibiting IκBα phosphorylation, CSG restricted the nuclear accumulation of NF-κB dimers, thereby suppressing NF-κB pathway activation (Figure 7B,C). The MAPK branch appeared insensitive to CSG treatment, as JNK, ERK, and p38 phosphorylation were not substantially affected, which was consistent with our luciferase reporter assay results (Figure 6A,D). These results indicate that CSG suppresses inflammatory responses in LPS-activated macrophages mainly by modulating the NF-κB pathway.

In fact, the in vivo anti-inflammatory actions of CSG have been determined in several models, including rhubarb oil-induced ear swelling, formaldehyde-caused paw edema, as well as acetic acid-increased permeability of capillaries [36]. To align with the in vitro model (LPS-activated macrophages), the in vivo action of CSG was determined in mice with endotoxemia. Unexpectedly, it indeed reduced serum IL-6, MCP-1, and TNF-α levels (Figure 2A–C). Notably, CSG failed to reduce the supernatant TNF-α level in LPS-stimulated macrophages (Figure S1 in Supplementary Materials). Such discrepancies indicate that the reduction in circulating TNF-α may not be solely attributable to effects on macrophages. In fact, during endotoxemia, circulating cytokines can be jointly contributed by multiple immune cell types, including hematopoietic immune cells and tissue-resident inflammatory cells [37,38]. Their serum levels are also influenced by microenvironments [39], which are more intended to be the reflective of an integrated response.

## 5. Conclusions

This study demonstrated that CSG showed inhibitory activity against LPS-induced inflammation in RAW264.7 macrophages and endotoxemic mice. In LPS-activated RAW264.7 macrophages (M1 cells), it inhibited the activation of NF-κB pathway by blocking p65 nuclear translocation, thereby suppressing the transcriptional and translational expression of iNOS, IL-6, MCP-1, and IL-1β. In the endotoxemic mouse model, CSG effectively reduced LPS-elevated pro-inflammatory mediators in serum, supporting its in vivo anti-inflammatory potential. Given the crucial role of macrophages in inflammation [1], the clinical potential of CSG in inflammatory diseases warrants further exploration—beyond merely serving as a substitute for “She Gan”, an identified anti-inflammatory traditional Chinese medicine [40].

## 6. Limitations

The present study linked the anti-inflammatory action of CSG to suppression of NF-κB signaling, but the upstream targets remain to be further explored and validated. Moreover, chemical composition analysis of CSG remains insufficient. Although the four identified components (tectoridin, iridin, tectorigenin, and irigenin) provide a plausible chemical basis for the observed activity, they collectively accounted for less than 23% of the total extract, making it difficult to fully represent the crude material. In future studies, more constituents (e.g., compound content markers for quality control) need to be identified, and pharmacokinetic data based on these markers should also be obtained. Moreover, given the important role of macrophage polarization in the inflammatory process, whether CSG can influence macrophage polarization warrants further investigation.

## Figures and Tables

**Table 1:** Sequences of primers used in the *q*RT-PCR analysis.

Gene	Primer	Sequences	References
*GAPDH*	F	5′-GGT TGT CTC CTG CGA CTT CA-3′	[22]
	R	5′-TGG TCC AGG GTT TCT TAC TCC-3′	
*iNOS*	F	5′-CTC AGC CCA ACA ATA CAA G-3′	[22]
	R	5′-CTA CAG TTC CGA GCG TCA-3′	
*IL-6*	F	5′-CTG CAA GAG ACT TCC ATC CAG-3′	[19]
	R	5′-AGT GGT ATA GAC AGG TCT GTT GG-3′	
*MCP-1*	F	5′-GCCCCACTCACCTGCTGCTACT-3′	[22]
	R	5′-CCTGCTGCTGGTGATCCTCTTGT-3′	
*IL-1β*	F	5′-GCAACTGTTCCTGAACTCAACT-3′	[19]
	R	5′-ACTTTTTGGGGTCCGTCAACT-3′	

## Data Availability

The data presented in this study are available on request from the corresponding authors. The raw data supporting the conclusions of this article will be provided upon request.

## Supplementary Materials

The following supporting information can be downloaded at: https://www.mdpi.com/article/10.3390/biomedicines14061291/s1, Figure S1: Effect of CSG on supernatant TNF-α in LPS-stimulated RAW264.7 macrophages (*n* = 3). ^##^ *p* < 0.01 vs. normal control group; ** *p* < 0.01 vs. LPS alone group.

## Abbreviations

The following abbreviations are used in this manuscript:

AP-1	activator protein 1
BC	*Belamcanda chinensis* (L.) DC
CMC-Na	sodium carboxymethyl cellulose
CSG	Chuan She Gan ethanolic extract
ELISA	enzyme-linked immunosorbent assay
ERK1/2	extracellular signal-regulated kinase 1/2
HPLC	high-performance liquid chromatography
IACUC	Institutional Animal Care and Use Committee
IL-1β	interleukin 1 beta
IL-6	interleukin 6
IMPLAD	Institute of Medicinal Plant Development
i.g.	intragastric (oral gavage)
iNOS	inducible nitric oxide synthase
IκBα	inhibitor of κB alpha
ITM	*Iris tectorum* Maxim.
JNK	c-Jun N-terminal kinase
LPS	lipopolysaccharide
MAPK	mitogen-activated protein kinase
MCP-1	monocyte chemoattractant protein 1
MyD88	myeloid differentiation primary response 88
NF-κB	nuclear factor kappa B
NO	nitric oxide
p38	p38 mitogen-activated protein kinase
p65	NF-κB p65 subunit
*q*RT-PCR	quantitative reverse transcription polymerase chain reaction
RAW264.7	murine macrophage cell line RAW264.7
SDS-PAGE	sodium dodecyl sulfate-polyacrylamide gel electrophoresis
TBST	Tris-buffered saline with Tween 20
TLR4	toll-like receptor 4
TNF-α	tumor necrosis factor alpha
