# Supplementary material for: Iridis tectori Rhizome Alleviates LPS-Triggered Inflammatory Responses Through Inhibiting NF-κB Signaling in Macrophages
Source: Biomedicines. 2026 Jun 5;14(6):1291. doi: 10.3390/biomedicines14061291 (PMC13297291; doi:10.3390/biomedicines14061291)

Figure. 3B iNOS

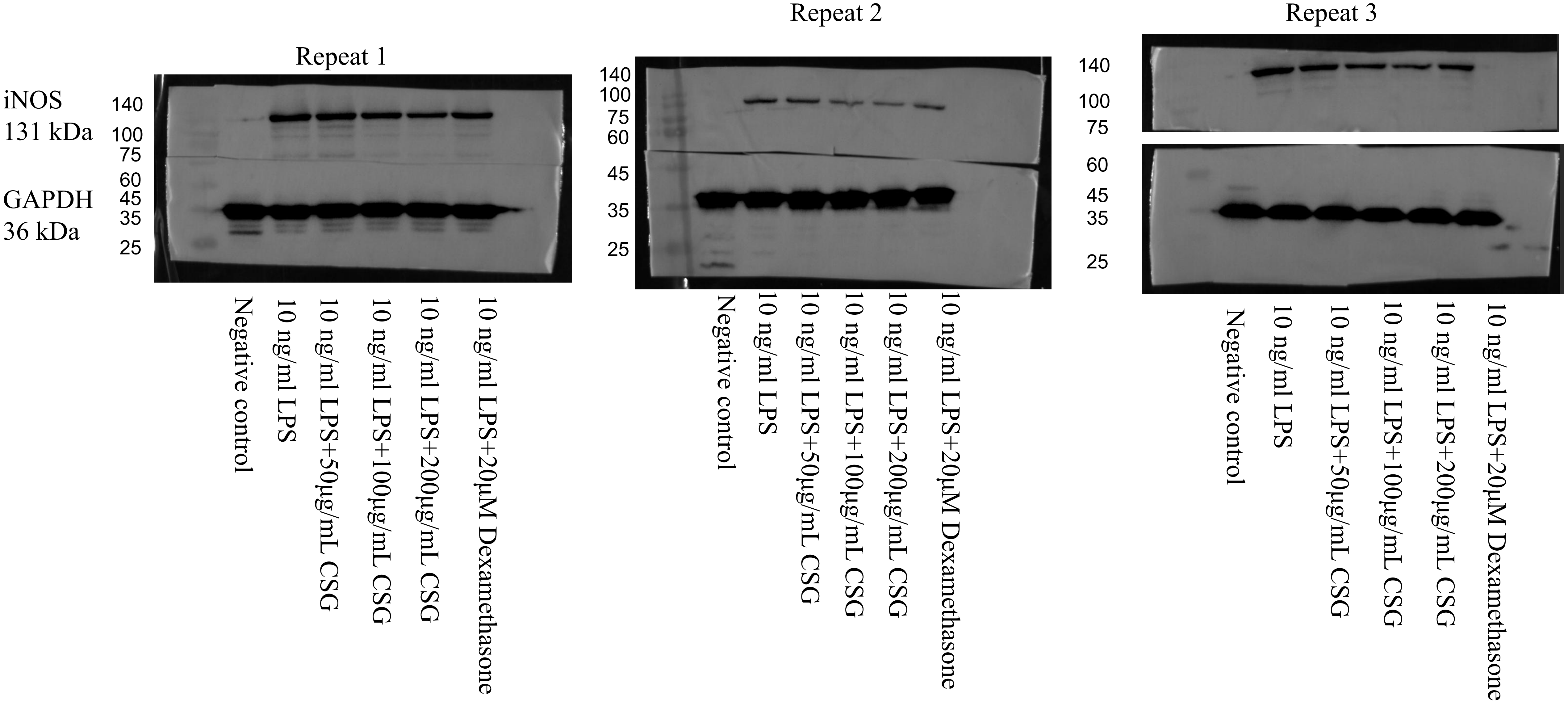

Figure. 5B p-ERK

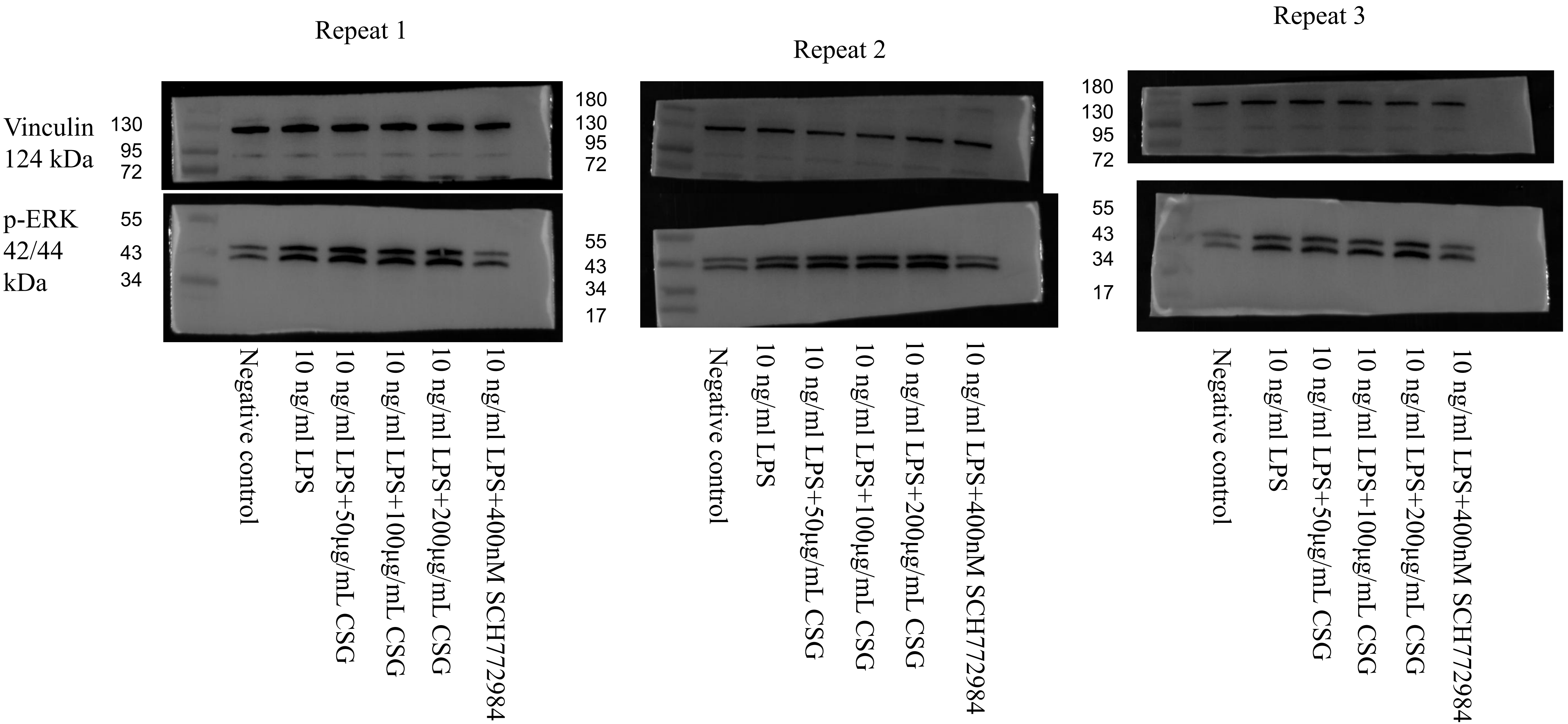

Figure. 5C p-JNK

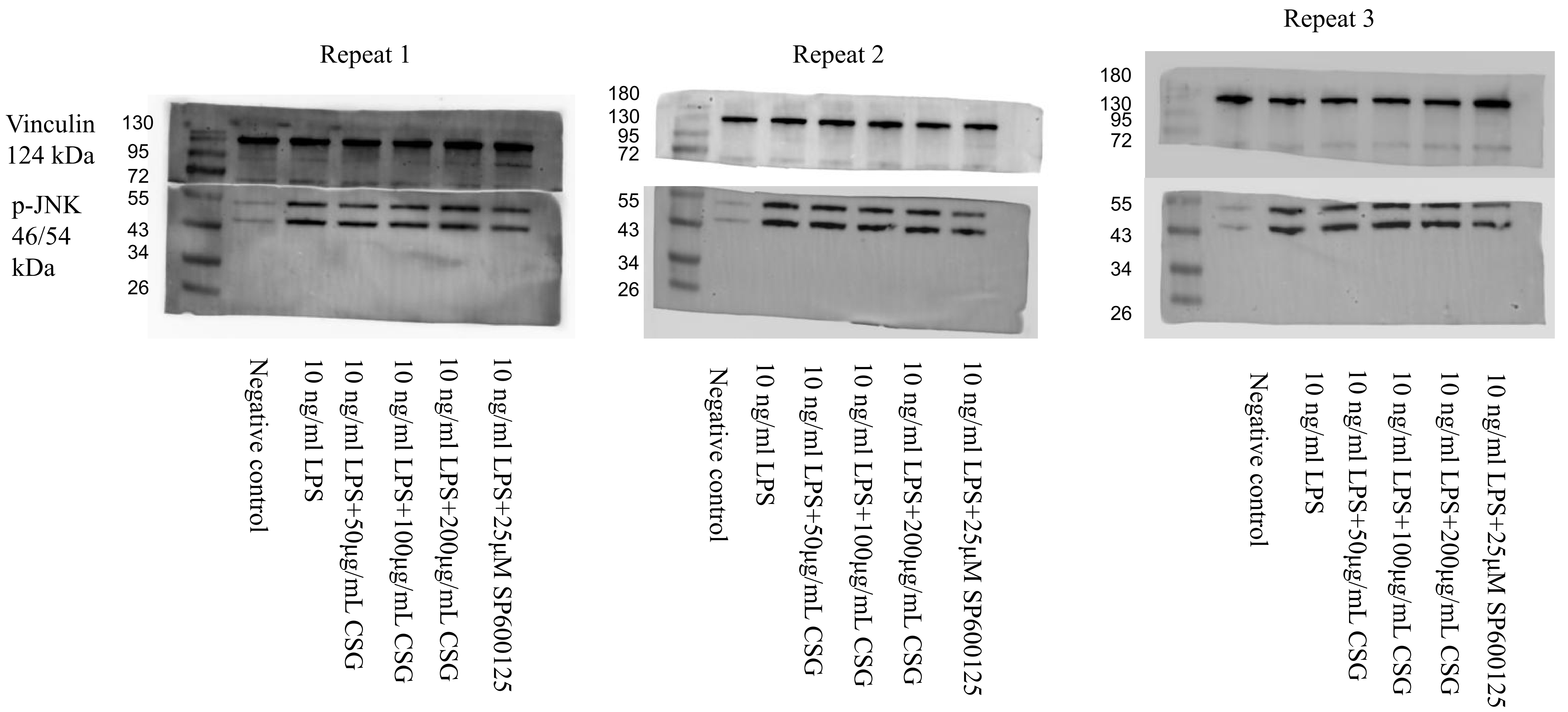

Figure. 5D p-p38

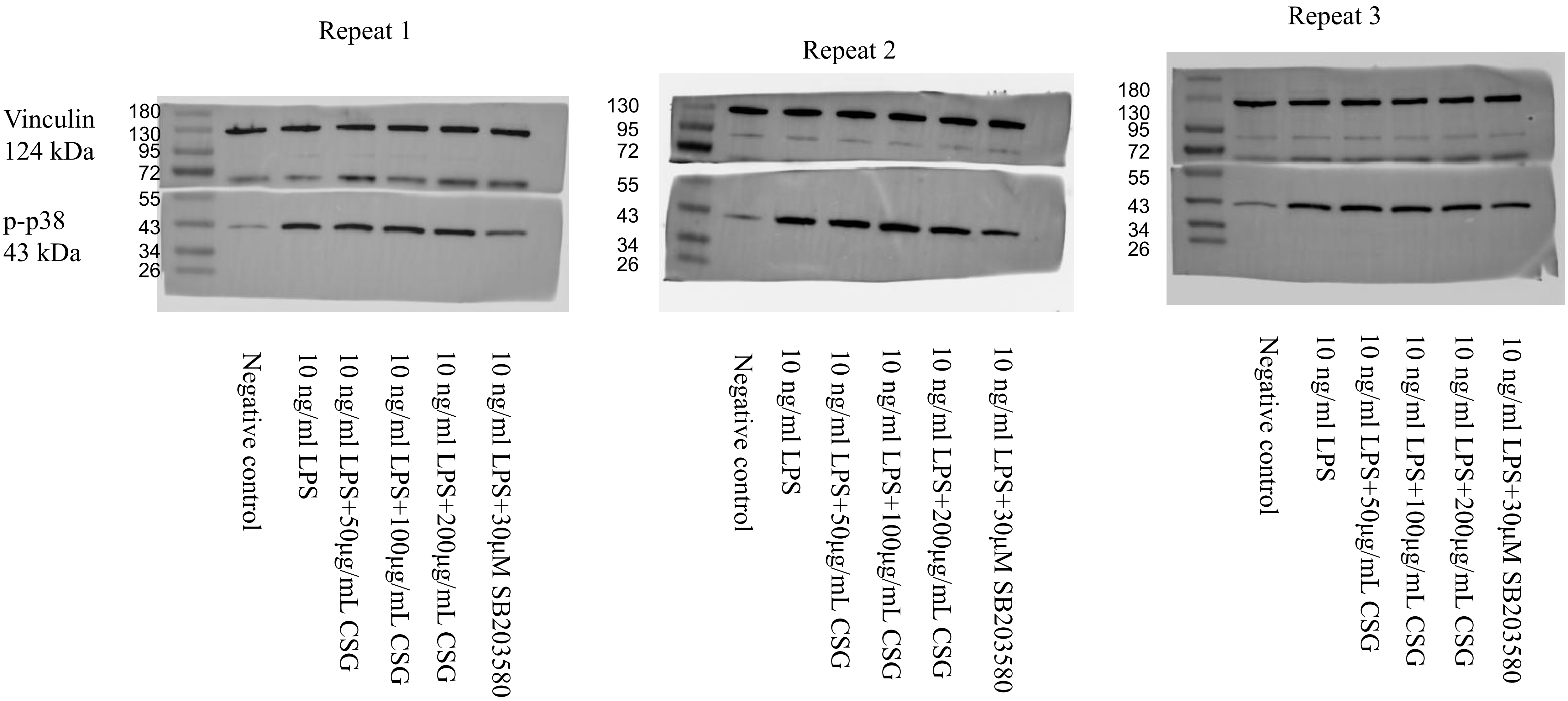

Figure. 6B IκBα

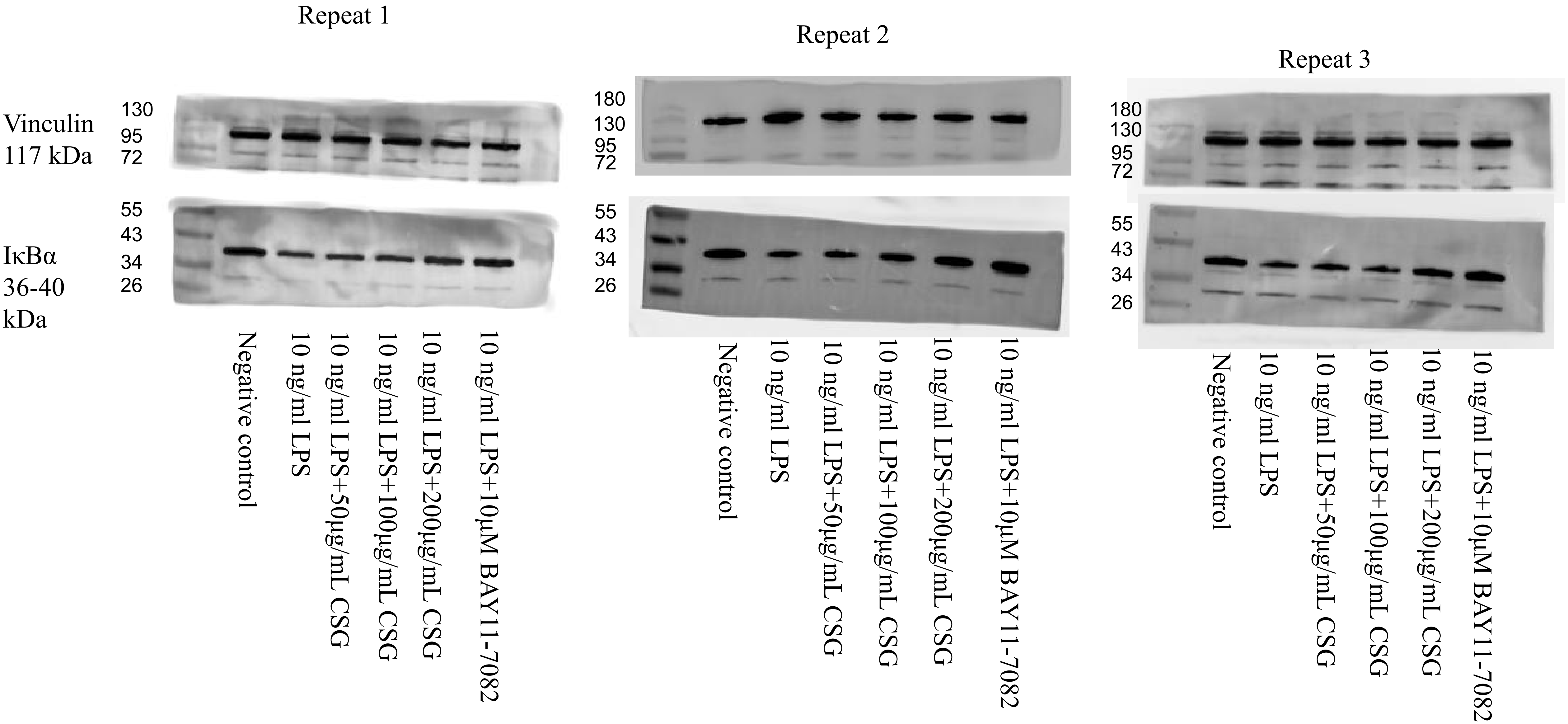

Figure. 6B p-IκBα

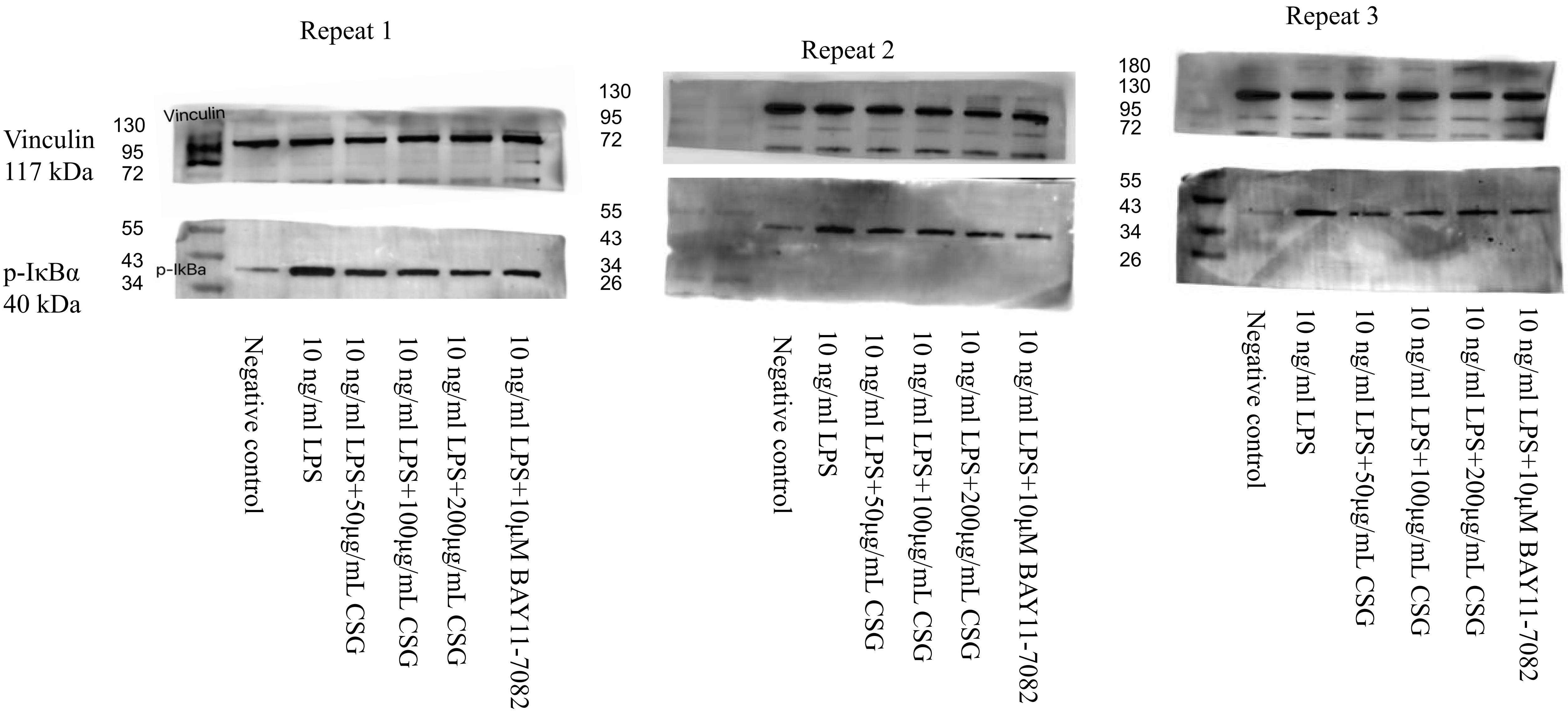

Figure. 6C Cytoplasmic

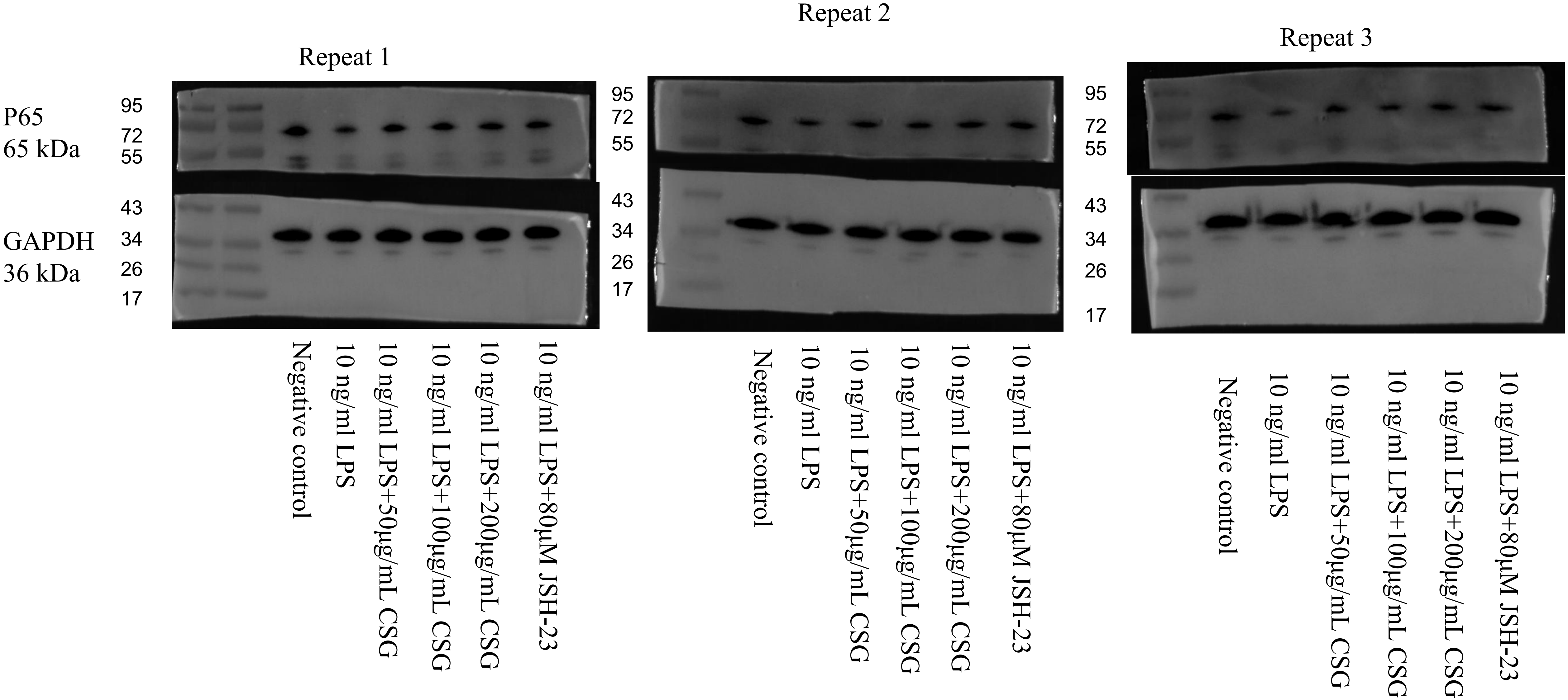

Figure. 6C Nuclear

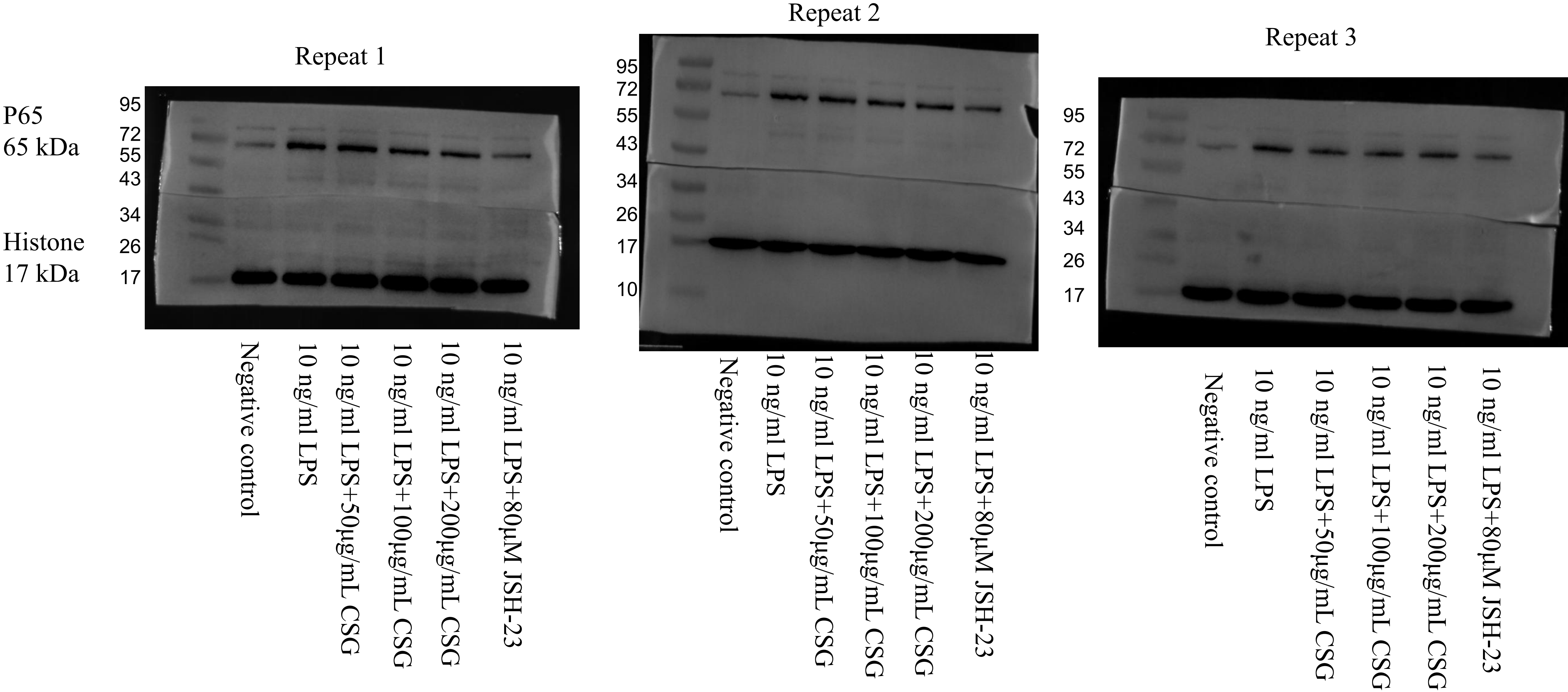

Supplement: Supplementary file 1 [file biomedicines-14-01291-s001.zip › Datas for wb images.pdf]
