# Supplementary material for: Iridis tectori Rhizome Alleviates LPS-Triggered Inflammatory Responses Through Inhibiting NF-κB Signaling in Macrophages
Source: Biomedicines. 2026 Jun 5;14(6):1291. doi: 10.3390/biomedicines14061291 (PMC13297291; doi:10.3390/biomedicines14061291)
Supplement: Supplementary file 1 [file biomedicines-14-01291-s001.zip › 5-21-supplementary files.pdf]

Supplementary file for

Iridis tectori Rhizome Alleviates LPS-Triggered Inflammatory Responses Through Inhibiting NF-κB Signaling in Macrophages

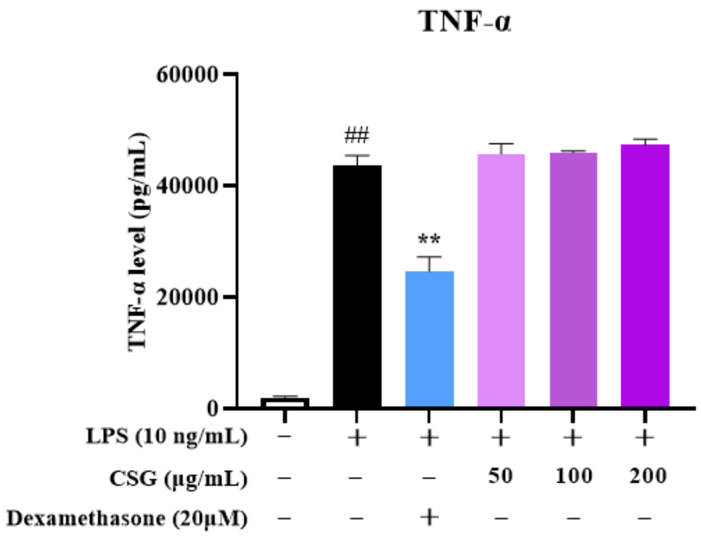

Figure S1: Effect of CSG on supernatant TNF-α in LPS-stimulated RAW264.7 macrophages (n = 3). $^{##}P < 0.01$ *vs.* normal control group; $^{**}P < 0.01$ *vs.* LPS alone group.
